# MITE Tracker: an accurate approach to identify miniature inverted-repeat transposable elements in large genomes

**DOI:** 10.1186/s12859-018-2376-y

**Published:** 2018-10-03

**Authors:** Juan Manuel Crescente, Diego Zavallo, Marcelo Helguera, Leonardo Sebastián Vanzetti

**Affiliations:** 1 Grupo Biotecnología y Recursos Genéticos, EEA INTA Marcos Juárez, Ruta 12 km 3, 2580, Marcos Juárez, Argentina; 2 Instituto de Biotecnología, CNIA, Instituto Nacional de Tecnología Agropecuaria (INTA) Castelar, Los Reseros y Nicolas Repeto, Hurlingham, Buenos Aires, Argentina; 3 Consejo Nacional de Investigaciones Científicas y Técnicas (CONICET), Buenos Aires, Argentina

**Keywords:** Transposable element, MITE, Tracker, Rice, Wheat

## Abstract

**Background:**

Miniature inverted-repeat transposable elements (MITEs) are short, non-autonomous class II transposable elements present in a high number of conserved copies in eukaryote genomes. An accurate identification of these elements can help to shed light on the mechanisms controlling genome evolution and gene regulation. The structure and distribution of these elements are well-defined and therefore computational approaches can be used to identify MITEs sequences.

**Results:**

Here we describe MITE Tracker, a novel, open source software program that finds and classifies MITEs using an efficient alignment strategy to retrieve nearby inverted-repeat sequences from large genomes. This program groups them into high sequence homology families using a fast clustering algorithm and finally filters only those elements that were likely transposed from different genomic locations because of their low scoring flanking sequence alignment.

**Conclusions:**

Many programs have been proposed to find MITEs hidden in genomes. However, none of them are able to process large-scale genomes such as that of bread wheat. Furthermore, in many cases the existing methods perform high false-positive rates (or miss rates). The rice genome was used as reference to compare MITE Tracker against known tools. Our method turned out to be the most reliable in our tests. Indeed, it revealed more known elements, presented the lowest false-positive number and was the only program able to run with the bread wheat genome as input. In wheat, MITE Tracker discovered 6013 MITE families and allowed the first structural exploration of MITEs in the complete bread wheat genome.

**Electronic supplementary material:**

The online version of this article (10.1186/s12859-018-2376-y) contains supplementary material, which is available to authorized users.

## Background

Transposable elements (TEs) or mobile DNA are distinct elements of DNA that move around within the host genomes by generating new copies of themselves into new chromosomal positions. TEs are abundant, ancient, and active components of genomes [1–3]. Because of their ability to transpose from one chromosomal location to another, which thereby increases their copy number, TEs can be major constituents in plant genomes and can act as drivers of genome evolution, expansion, and plasticity [3, 4]. Moreover, there is increasing evidence that TEs also play a key role in regulating gene expression and epigenetic modification[5].

By consensus [6], TEs are hierarchically organized, first, in two classes (the highest level) according to the presence or absence of an RNA transposition intermediate into RNA (class I or retrotransposons) or DNA (class II or DNA transposons), respectively. Then, they are classified in subclasses according to the mobility during the reverse transcription and the number of DNA strands cut at the donor site. The following hierarchy is order and depends on the insertion mechanism. Subsequently, they are organized in superfamilies by large-scale features such as the structure of protein or non-coding domains. Finally, they are classified in families and subfamilies according to DNA sequence homology and conservation. A TE family is defined as a group of TEs with high DNA sequence similarity. In this classification system, two elements belong to the same family if they shared at least 80% of sequence identity in at least 80% of their coding or internal domain. The mentioned homology should produce strong BLAST hits at default settings [6]. Another widespread and similar classification system is proposed in Repbase [7]. According to this criterium, all eukaryotic TEs belong to two types (retrotransposons and DNA transposons) and can be further divided into classes according to enzymology, structural similarities and sequence relationships. Each TE class can be divided into a small number of superfamilies or clades and each superfamily, into numerous families (consensus sequences of any two families should be less than 75% identical) [8]. In both mentioned systems, class I TEs, or retrotransposons, transpose by making a new copy from the original element via RNA intermediates and pasting it into a new locus. Class II elements, or DNA TEs, cut out DNA of the original element (single strand or double strand) and paste it into the new locus. Finally, TEs can also be autonomous or non-autonomous depending on whether the enzyme required for transposition, which is known as transposase, is produced by itself or by a different TE [9]. In this work we focus on a group of non-autonomous class II TEs known as miniature inverted-repeat transposable elements (MITEs). MITEs are structurally characterized by their relatively small size (generally 50-800 bp long), high copy number and lack of coding capacity for transposases. They bear Terminal Inverted Repeats (TIRs) and two flanking short, direct repeats called Target Site Duplications (TSDs) [10]. MITEs are considered as truncated derivatives of autonomous DNA transposons and are grouped into super-families based on their association with those TEs because they have the same or very similar TIRs [11].

MITEs are often found close to or within genes and are involved in gene regulation. In wheat, a MITE insertion within genes or in their regulatory regions produces changes in their expression. For example, the insertion of a MITE in the promoter region of the *Vrn-A1* gene causes the deregulation, thus conferring the loss of vernalization requirements to flower [12].

Another pathway of gene regulation is through epigenetic silencing produced by microRNAs or siRNAs that are derived from MITE, which through their rapid rate of rearrangement or decay represent a constantly evolving source of new microRNA genes. A comparison of experimentally determined microRNAs with repeat databases revealed that 6.5% of *Arabidospsis* and 35% of rice microRNAs co-localized with TEs, most of them MITEs[13, 14].

The existing tools to find hidden MITEs in genomes use different methods and are classified in three major groups: de novo, homology-based and structure-based. The de novo method makes use of the intrinsic characteristics of MITEs such as repetition of mobile DNA in genomic sequences, usually without using structure information or similarities with known TEs. Another de novo approach is to use siRNAs that are a part of TEs silencing pathways as a guide and to map these elements into genomic sequences [15]. The homology-based method makes use of known TE sequences to find hidden MITEs. Even though tools based on this method are good at detecting real TEs, they cannot detect novel TEs [16]. The well-defined structure of a MITE makes it possible to elucidate putative elements by finding sequences that have a TIR and a TSD. This approach is known as the structure-based method. Only real MITEs are expected to have a certain copy number with different flanking sequences [16], because these features are indicators that the element was transposed into different genomic locations.

DetectMITE [16] has proven to be efficient in detecting candidates by using a complex-number-based numeric calculation to detect perfect and imperfect inverted repeats and using cd-hit [17] to identify clusters from sequence similarity. This open-source software package uses MATLAB as the programming language.

Another well-known program in this group is MITE Digger [18], a desktop tool that can detect MITEs in full genomes by using a computational strategy that processes a smaller portion of genome at a time. This program, however, has been proven to miss many cases [16]. A compiled version of MITE Digger is available in the developer’s webpage and can be used only under Microsoft Windows operating system (OS). Another program, MITE Hunter [19], can discover MITEs as well as other short non-autonomous Class 2 TEs in genomic data sets. MITE Hunter searches for TIR-like structures in genomic fragments. Then, it uses all-by-all BLAST search (BLASTN) to group similar elements into families and filtering low copy number candidates. All the mentioned programs can use genomes such as those of rice or *Arabidopsis* as input, but failed to process large genomes such that of bread wheat. Here we present MITE Tracker, a novel software program that, according to our results and comparisons, identifies MITEs with the best results to date in terms of false-positive rates and processing efficiency in complex genomes. MITE Tracker uses a fast and low-memory consuming algorithm to search for putative MITEs in genome sequences. Furthermore, a meticulous false-positive filtering criterium makes this tool the most accurate. The installation and execution of MITE Tracker is easy and straightforward in comparison to the other mentioned tools. The result files given by the program are easy to understand and use in downstream analysis.

## Materials and methods

### Genomic sequences

The rice genome (*Oryza sativa* Os-Nipponbare-Reference-IRGSP-1.0) [20] was used as a test case to compare MITE Tracker, detectMITE [16] and MITE Hunter [19]. Outputs were obtained by running each program with the same input data. The wheat genome reference assembly (IWGSC Ref Seq 1.0) was used to detect MITEs and to test the processing capabilities of the three software packages. The hexaploid (bread) wheat (*Triticum aestivum* L.; 2n = 6x = 42; genomes AABBDD) has a genome size of 14 Gbp [21]. This is 35 times larger than that of the Nipponbare rice genome (*Oryza sativa* L.; 2n = 2x = 24) (*Oryza sativa* L.; 2n = 2x = 24) [20] which is 389 Mbp. Wheat has more than 80% of its genome constituted of repetitive DNA[22]. The fact that MITE discovering algorithms rely on repetition makes bread wheat genome a challenging candidate for testing the processing capabilities and accuracy of this method.

### Programming language and testing environment

MITE Tracker runs under Python 3 and makes use of the programs NCBI Nucleotide-Nucleotide BLAST 2.6.0+ [23], VSEARCH 2.7.1 [24] and the Python libraries pandas 0.19.0 [25] and biopython 1.70 [26]. The mentioned programs are freely available open-source software. Exhaustive testing was done using an Ubuntu 16.04.1 machine with 64GB of RAM and 10 dedicated cores.

### Transposable elements databases

Repbase Update is a well curated database of transposable elements (TEs) and other types of repeats in eukaryotic genomes. Sequences from *Oryza Sativa* were downloaded from the web page in EMBL format [27]. Of 2734 elements, 569 were filtered using a python script. Only elements labeled as MITEs or Class II DNA non-autonomous TEs that were shorter than 801 nt were kept. This database is usually used as a reference when comparing transposable elements detection programs. In this case we used Repbase Update to evaluate accuracy of the compared programs. The Triticeae TEs database TREP database was used to classify MITE families in wheat [28].

## Implementation

### MITE Tracker algorithm

#### Identification of MITE candidates sequences

**Fig. 1:**
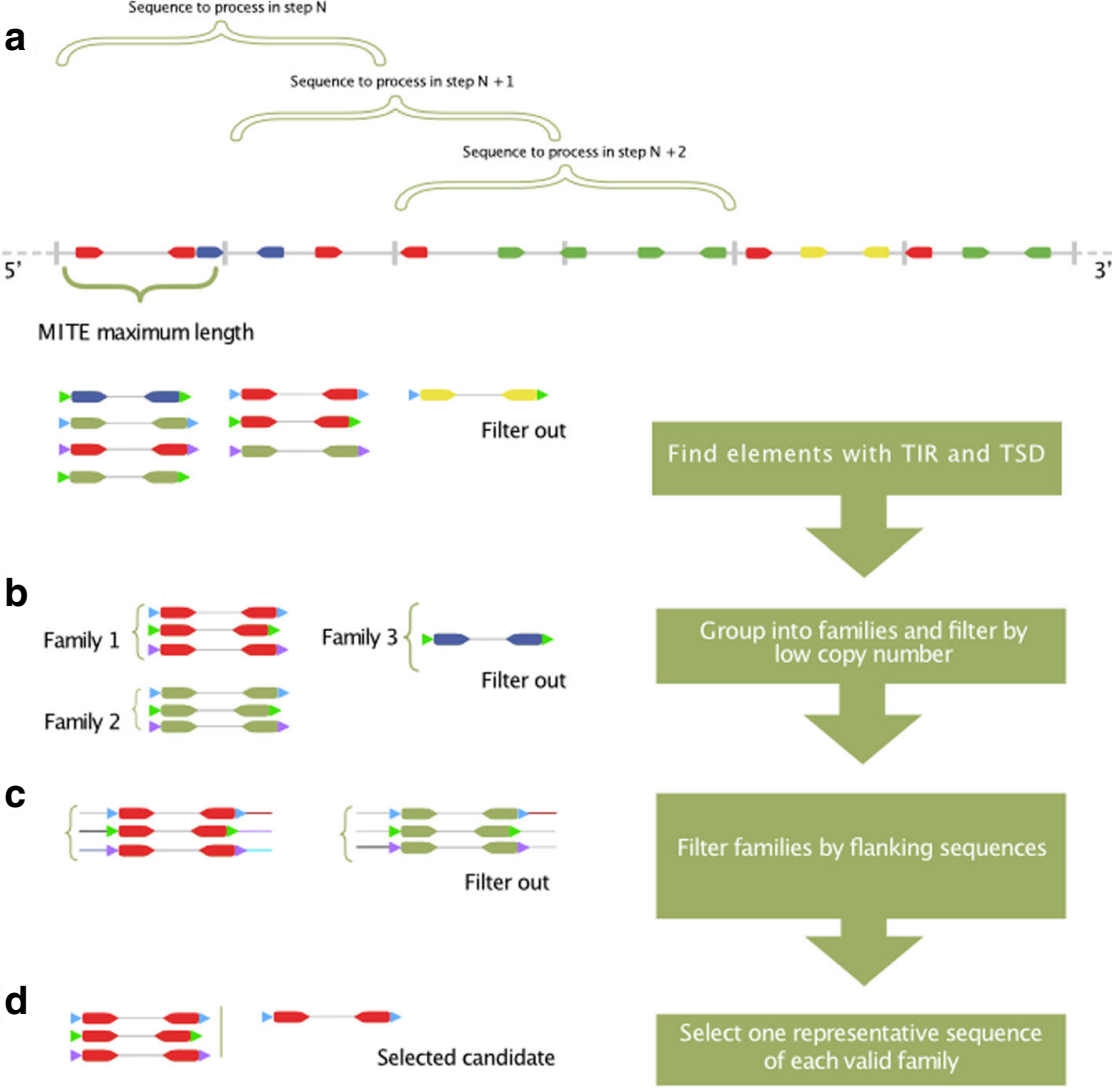
MITE Tracker algorithm

The first step to find MITEs hidden in genomes is to identify putative elements with a certain structure (a sequence with valid TIR and TSD). To find those candidates, MITE Tracker first searches for valid inverted repeat sequences of a given length (by default between 50 and 800 nt). It retrieves inverted repeats out of small pieces of the genome at a time by splitting the genome into segments and aligning each segment to its reverse complementary sequence. At this step, a nucleotide-nucleotide BLAST search is used to align the sequences, thus allowing TIRs to have mismatches and gaps among the alignment. Because the genome is processed in small segments, memory consumption remains low. This also allows the program to process several segments in parallel. According to a user defined parameter that specifies the MITE maximum length (MITE MAX LEN), genome sequences are divided into segments of twice this value for comparison. This is done to minimize unnecessary comparisons between sequences that are separated by more than the established MITE MAX LEN. Also, all the sections of the genome that could be part of two TIRs of the same MITE are compared to each other (see Fig. 1a).

For each segment, the first processing step consists of calculating a Local Composition Complexity (LCC) score. Highly repetitive sequences are considered simple (a lower value), whereas highly non-repetitive sequences are considered complex (a higher value) [29]. The LCC value is configurable by the user via the -lcc parameter. By default, the candidate sequence is required to have a complexity value of at least 1 to be processed; otherwise, it is discarded. This LCC value gives a low false positive rate, while still considering real MITEs according to our tests. A BLAST search is used to compare the segment and its reverse complementary. The obtained alignments of TIRs must have a length of at least 10 nt (by default). Elements whose terminal sequences have very high or low GC content are usually detected as an inverted repeat by this algorithm. For this reason, the GC content must be between 15% and 95%. A complexity score is calculated again for the putative MITE sequence and only LLC values equal or higher than 1 are kept. For the next step, the starting position of the next segment to be processed is incremented by MITE MAX LEN. As mentioned above, this will overlap the current sequence with the previous one by a span of 50%, thus making sure that all the putative TIRs are retrieved. After finding a valid TIR pair, the left and rightmost positions are extended to check whether TSDs exists. Before adding a sequence to the candidate list, the program checks whether the element is nested inside another. Two elements are considered nested if the TIR of one of the elements overlaps with the TIR of the other. This means that the two found candidates are the same MITE and discards the possibility of being two different MITEs, one inside another. In the case of overlapping, only the longest sequence is saved and the other candidate is discarded. If all these requirements are fulfilled, then the sequence is saved as a valid candidate (see Fig. 1a).

#### Clustering sequences into families

Correctly retrieving similar sequences is a crucial step in MITE identification because these elements are presented in high copy number and because computing time and space usage for clustering is usually expensive. We propose the advantages of VSEARCH to perform such operation. This tool uses a greedy and heuristic centroid-based algorithm with an adjustable sequence similarity threshold (see Fig. 1b). TSD sequences are removed at this point to compare internal sequences, because they can change upon different insertions. VSEARCH is executed with parameters –iddef 1 and -id 0.8. This means it uses a BLAST-like distance calculation and a similarity of 80% for clustering [24].

#### Flanking sequence filtering

For each element, right and left flanking sequences (sequences surrounding the element outside the TSD, by default 50nt length) are retrieved and compared with the flanking sequences of all other elements of the same family using a local alignment algorithm. This is done to check if the element was transposed into different genomic locations. When a MITE is transposed, it is less likely that its flanking sequences will also be transmitted together [16]. Comparisons are done between each pair of flanking sequences of putative MITEs of the same family using a local pairwise alignment: Left flanking sequence of one against the other, right against right, reverse complement of right against left and reverse complement of left against right. Furthermore, two members of a cluster may differ slightly in length and in the TIR sections. In this case, part of the flanking sequence of a member may be partially inside other member. To overcome this putative scenario, the program also compares the flanking sequences to the internal sequence of other elements within the same family. The program performs eight comparisons for each pair of members in each cluster. Table 1 shows the comparisons required between two putative elements of the same family. Only elements that differs completely in the flanking sequences against all other elements in the same family are considered to be different individuals of the same MITE family. When all elements of the family are processed, the family is conserved only if the number of different individuals is equal or above a user-defined minimum copy number threshold (3 by default, see Fig. 1c).

**Table 1:** Comparisons done between flanking sequences

1	Flanking sequence right	Flanking sequence right
2	Flanking sequence left	Flanking sequence left
3	Flanking sequence left reverse-complemented	Flanking sequence right
4	Flanking sequence right reverse-complemented	Flanking sequence left
5	Flanking sequence right	Flanking sequence right + full MITE
6	Flanking sequence left	Flanking sequence left + full MITE
7	Flanking sequence left reverse-complemented	Flanking sequence right + full MITE
8	Flanking sequence right reverse-complemented	Flanking sequence left + full MITE

#### Representative sequence selection

Because VSEARCH cluster algorithm does not automatically provide a representative sequence for each cluster and, for this reason, the first reported sequence of each family is selected arbitrarily. From all the elements in a family, the most common TSD, if there is one, is indicated in the family (see Fig. 1d).

#### Running MITE Tracker

MITE Tracker is an open source multi-platform that has been tested on Linux (Ubuntu and Debian), macOS High Sierra and Windows 10. The source code is available at https://github.com/INTABiotechMJ/MITE-Tracker. A quick setup and running guide is provided in the repository. It is important to remark that MITE Tracker is developed under Python 3 programing language, which makes it easy to install and run for users with limited knowledge of command line programs. The fact that it runs in different platforms (Windows, Linux, MacOS) is also important when users want to try the program on their own personal computers.

## Results

### Rice genome

**Fig. 2:**
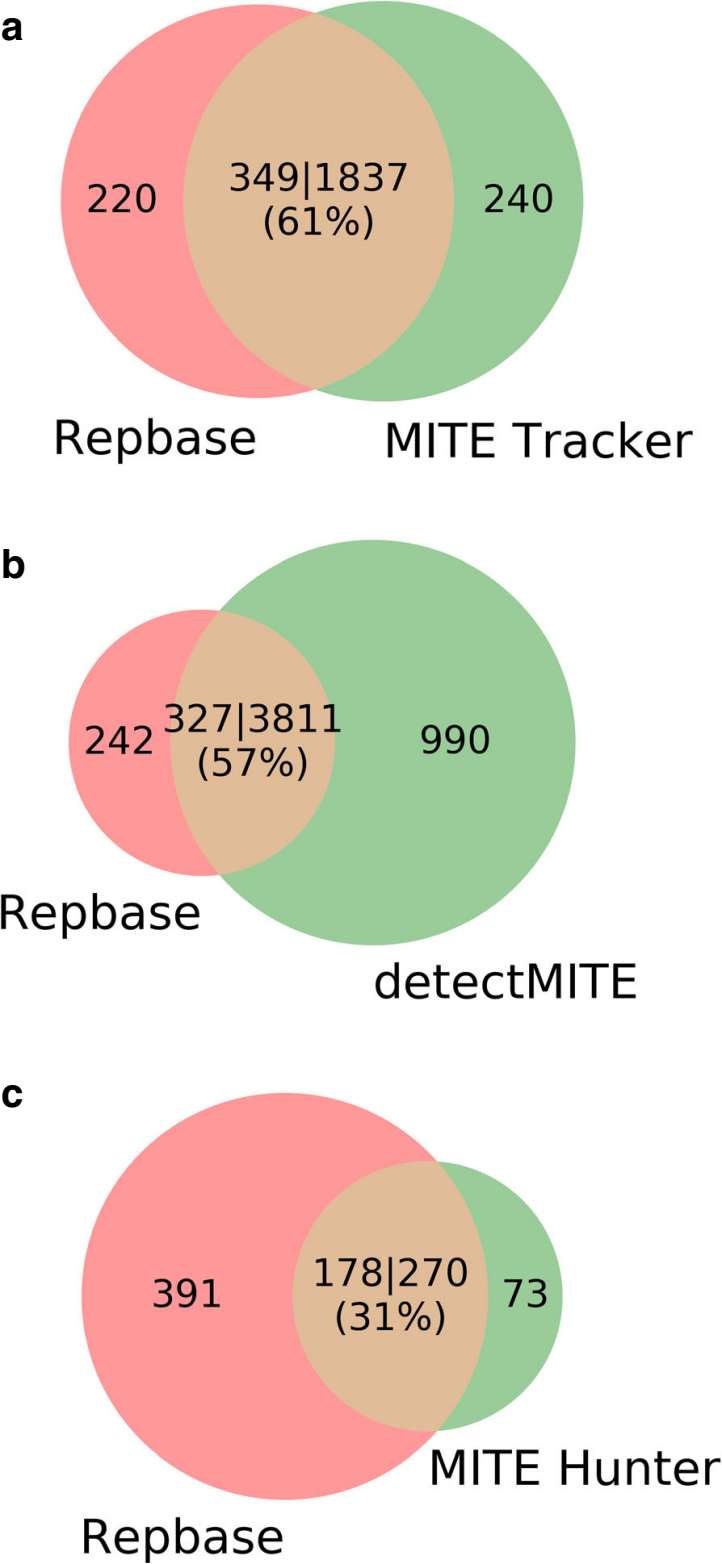
Coverage of repbase *Oryza sativa* non-autonomous elements from three different programs **a** Comparison of MITE Tracker vs repbase **b** Comparison of detectMITE vs Repbase **c** comparison of MITE Hunter vs Repbase. Using **a** as an example, the number 317|1549 means that 1549 elements of MITE Tracker matches 317 elements of Repbase covering a 55%

Rice was used as the input genome to test MITE Tracker, detectMITE and MITE Hunter. All three programs used 10 threads simultaneously for this experiment. MITE Tracker detected 17,651 full MITE in the rice genome (see Table 2) in 2.5 h and only 20 of these elements (0.13%) were nested in larger elements. The clustering process classified them into 2077 different families. Two versions of Repbase were used as a reference database: one containing all the TEs of *Oryza sativa* (total Repbase) and other only with putative MITEs, non-autonomous elements shorter than 801 bp (filtered Repbase). The three programs tested here were expected to find only valid MITEs with a length between 50 and 800 bp. MITE Tracker’s output hits 349 elements of Repbase using 1837 families (Fig. 2b). The elements with a match in total Repbase but not in filtered Repbase are considered false positives. Of the 1837 matches against filtered Repbase, 81 (4%) hit only the total version of the database. DetectMITE took 28 h to process the whole rice genome and detected 36,029 elements, of which 10,069 (28%) are nested between each other. These elements are grouped into 4801 families. They match 721 elements of total Repbase using 4549 families and 327 of filtered Repbase by using 3811 families. Of the 4549 families, 741 (16%) are false positives.

**Table 2:** Comparison of MITE Tracker, detectMITE and MITE Hunter with the rice genome as input

	MITE tracker	DetectMITE	MITE hunter
Processing time	2.5 hs	7 hs	40 hs
Total elements	17,651	36,029	-
Nested elements	20 (0.13%)	10,069 (28%)	-
Total families	2077	4801	343
Filtered Repbase matches^a^	349 (61%)	327 (57%)	178 (31%)
False positives^b^	81 (4%)	741 (16%)	25 (8%)

^a^Repbase filtered by non-autonomous elements and less than 801 bp ^b^Elements from each program output that are TEs and not MITEs according to Repbase

**Fig. 3:**
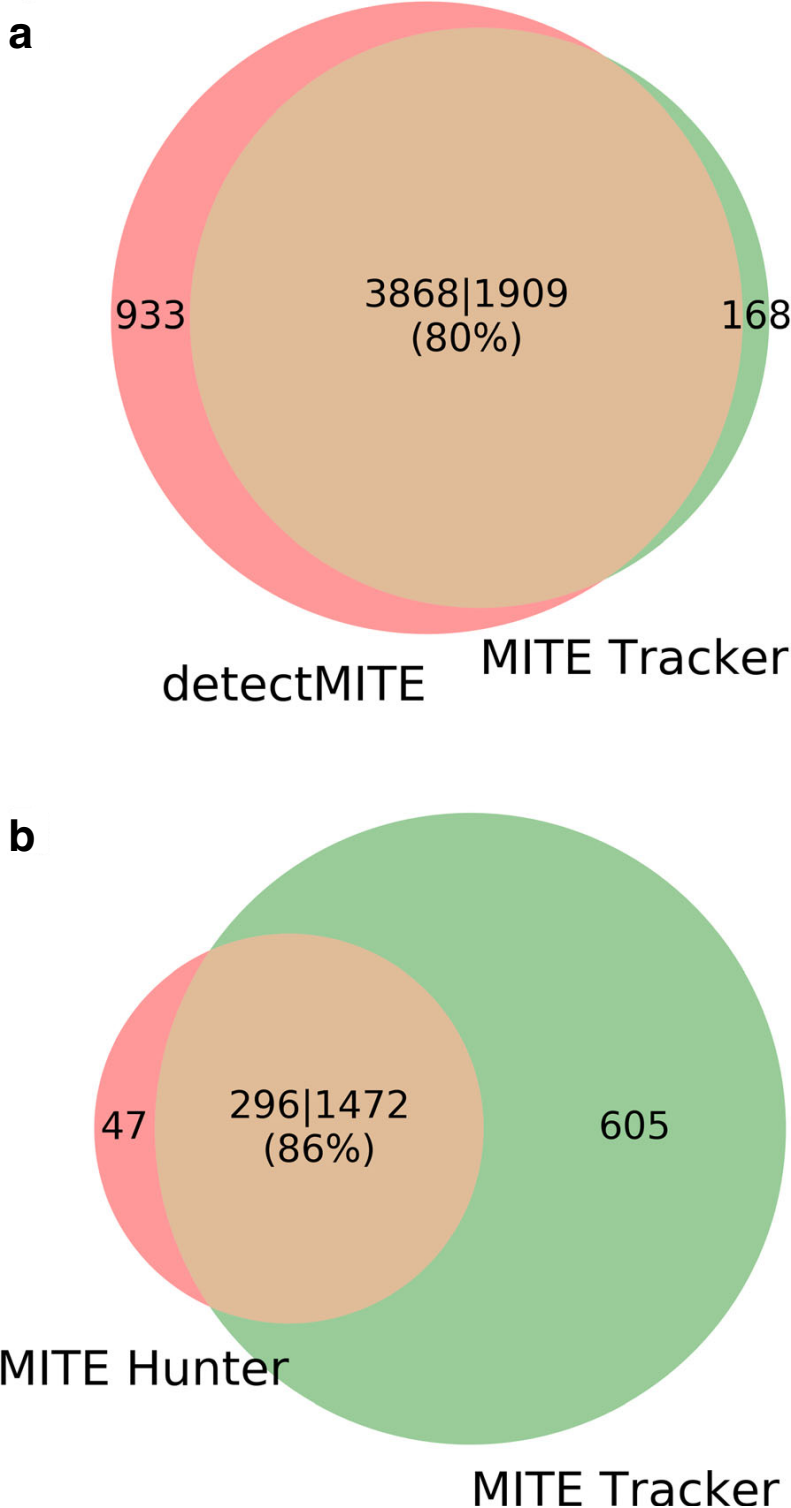
Coverage of other program’s output from MITE Tracker **a** Comparison of MITE Tracker vs detectMITE **b** Comparison of MITE Tracker vs MITE Hunter

The families of detectMITE that are not covered by MITE Tracker (990) (Fig. 3a) are all contained in the initial candidates of MITE Tracker. In a further analysis, we observed that these families are mostly discarded by flanking sequence comparison.This occurs because, unlike detectMITE, MITE Tracker compares them in both strands using a reverse complementary sequence, unlike detectMITE.

MITE Hunter took 40 h to find 343 families. Of these 343 elements, 295 have significant hits against 247 elements of Repbase (Fig. 2c), and 270 significant hits against 178 elements of filtered Repbase. False positive is 25 (8%). The elements obtained by MITE Hunter cannot be classified as nested or not nested, because they lack genome coordinates in the description. Furthermore, MITE Tracker can retrieve 80% and 86% of the elements discovered by detectMITE and MITE Hunter, respectively (Fig. 3a). Table 2 shows the results obtained from the three compared software packages. MITE Tracker overcomes the other two in terms of performance and accuracy.

A Tourist-like MITE sequence of 430 base pairs known as mPing, which transposes actively in *Oryza sativa* L. ssp. indica cell-culture line [30],was used as a reference to conduct a BLAST search with outputs from the three software. MITE Hunter gives no results, thus showing that it misses this element. Although DetectMITE did found this element, the sequence is found three times in three different families. By contrast, MITE Tracker successfully identifies mPing 48 times in the same family. Some (240) of the elements found by MITE Tracker are not listed in Repbase. These elements may be newly detected MITE families but we cannot discard that some of them are false positive (4% of false positives according to our experiments).

### Running the giant wheat genome

MITE Tracker took about 10 days of computing time to run bread wheat entirely. Chromosomes can be run separately on different computers and the clustering process can be executed with merged results (instructions are available in source code). A minimum copy number of four elements was used to obtain 6013 families (available as Additional file 1), which were formed by 128,453 complete elements. The two other programs failed to load the wheat genome and thus produced no output. Using MITEs families as input, we conducted a BLAST search to find MITE elements on a genome wide scale with a similarity constraint of at least a 95% coverage of the input sequences and at least 95% identity between query and target sequences. The search retrieved 682,397 elements in the wheat chromosomes, in average 48 MITEs per Mb. Interestingly, of 110,790 genes in the wheat sequences, 7766 (7%) have a MITE insertion. The genome coverage gives 0.16% of the genome covered with MITEs with the selected level of homology and coverage in the blast searches.

**Fig. 4:**
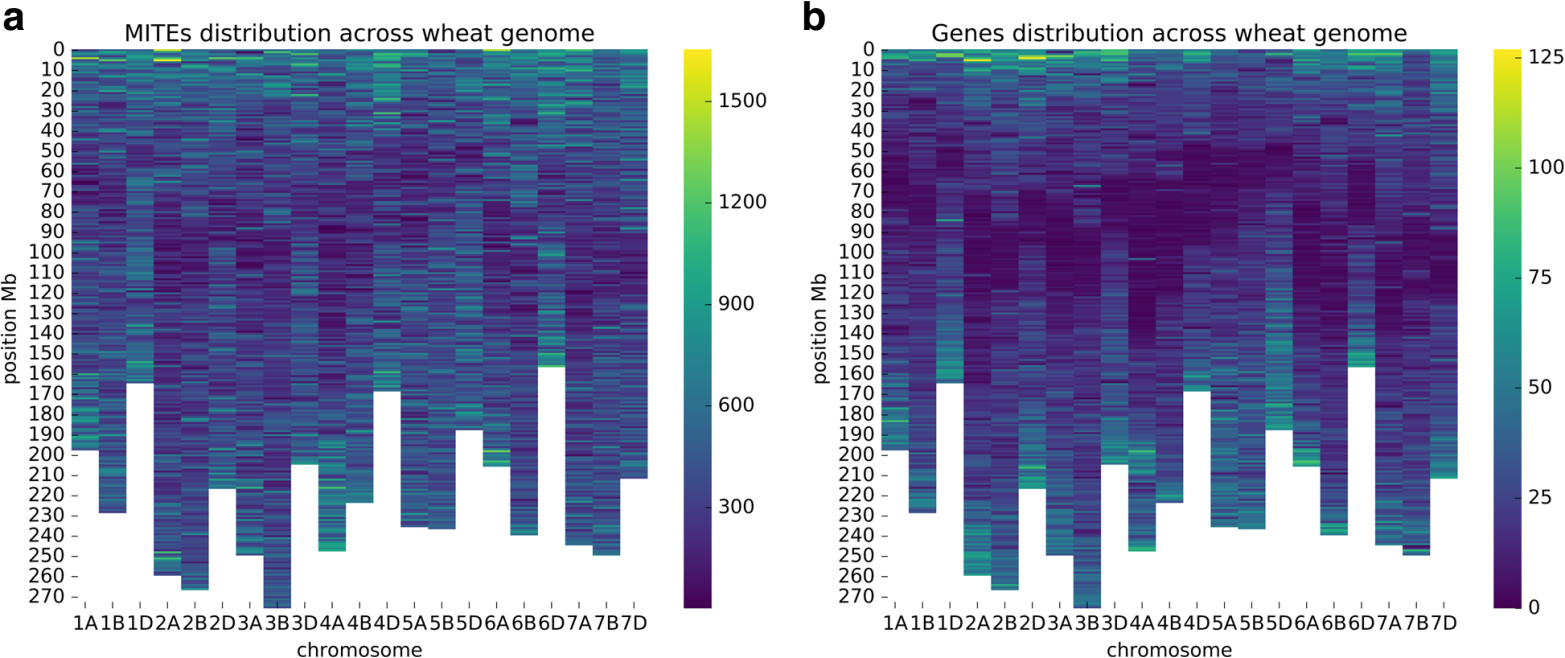
**a** shows distribution of MITEs across wheat genome in a 3 Mb resolution. **b** shows distribution of genes with the same schema

**Fig. 5:**
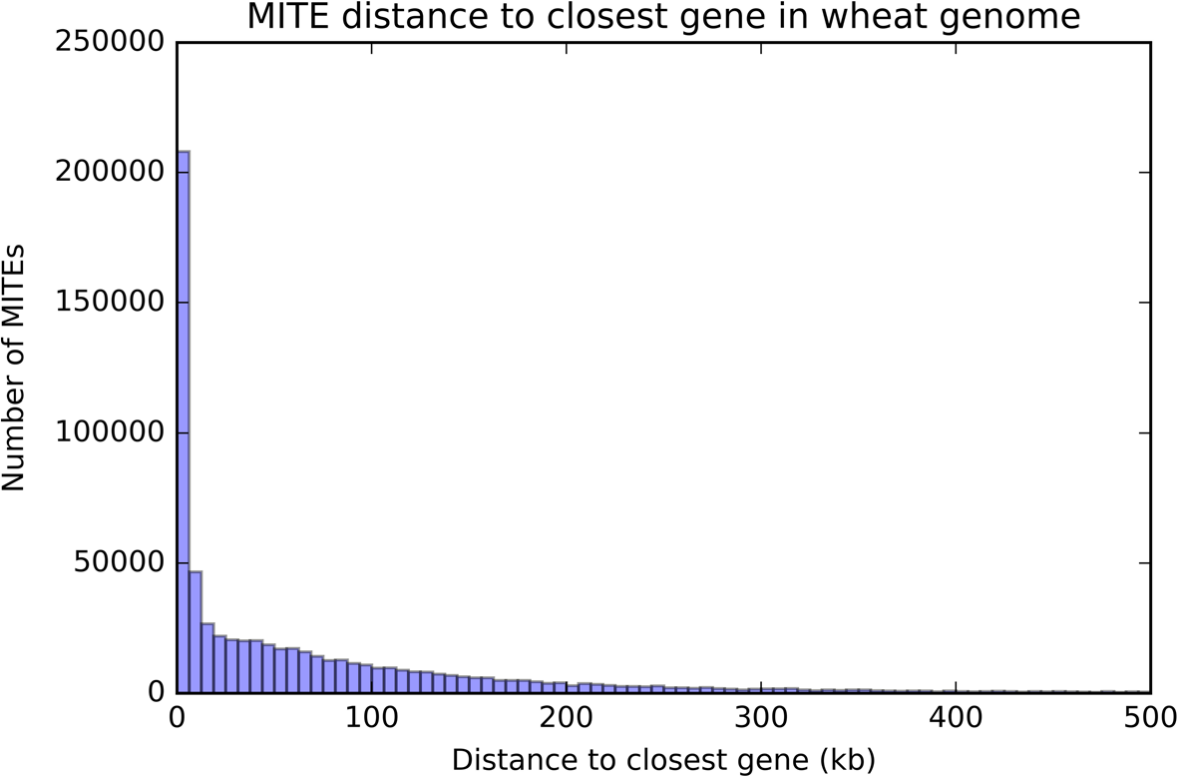
Number of MITEs by distance to closest gene

MITEs are distributed along wheat chromosomes and correlate with gene-rich regions (Fig. 4a). The highest densities of these transposons are located in the telomeric regions, which correlates with the high density of genes (Fig. 4b). Figure 5 shows a histogram that represents how MITEs are distributed along genes. MITEs are likely to be within or close to a gene in the wheat genome.

### Running other genomes

No MITE was detected in the genome of the ultrasmall unicellular red alga Cyanidioschyzon merolae. The same result was previously obtained in other research [31]. In this study, the Plasmodium falciparum genome also reported no MITEs as expected and in accordance with other previous studies in which no transposable elements or retrotransposons were identified [32].

Additionally, as another control, we generated a random genome from the rice genome, divided it into 6-mers and randomly shuffled and concatenated these 6-mers to produce a shuffled genome. Finally, we removed the repeats found by RepeatMasker [33] in the shuffled genome. This repeat-free random genome reported no MITE by MITE Tracker. Any result found in the random genome would be considered false positives. *Solanum Tuberosum* [34] was also used to compare the performance of MITE Tracker against other tools. Execution time and number of results of this and other genomes are available in Additional file 2: Table S1. Empty cells indicate that the program is unable to handle input genome.

## Discussion

### MITE Tracker methodology

New tools for the identification and annotation of TEs emerge regularly, mainly since the functional role of these elements in genome evolution and transcriptional regulation has been discovered. A correct detection of these elements, however, is still a bottleneck of the available tools and highly depends on the type of TEs. Because of the specific characteristic of MITEs, the structure-based method is very reliable for MITE discovery. Nevertheless, most of these methods retrieve high rates of false positives, are unable to correctly discriminate families and fail to process large genomes.

Implementing clustering methods such as cd-hit and all-by-all BLAST in a large set of MITE candidate sequences is, in many cases, prohibitive in terms of execution time and memory usage. VSEARCH [24] is used for clustering similar sequences into families in MITE Tracker. The use of VSEARCH accelerates the clustering step and makes it more efficient. Therefore, the use of this tool together with MITE Tracker allows the processing of huge genomes with a reasonable execution time.

Two MITEs candidates are considered nested if one of them is inside another and the TIR section overlaps partially between them. DetectMITE detects a large number (28%) of nested MITEs. Because these elements are clustered together to form families, the results given by detectMITE have many duplicated sequences. Furthermore, some of the totally nested elements only differ in one base at the beginning and one at the end and, despite of being almost identical sequences, they are grouped into different families (data not shown). In this scenario, detectMITE output contains families that consist of many cases of recurrent elements. If nested elements were removed from the candidates, the family number would decrease significantly because they would not reach the minimum copy number elements per family. This could also decrease the percentage of false positive in this analysis. MITE Tracker uses a different approach to overcome situations when nested TIRs are found. Before adding a candidate, this program checks that there is no other element in the same position containing the current candidate (the TIR sequences do not overlap). In the case that a previous element is inside the new element, the program deletes the previous element and add the new one (always keeping the larger element). This approach reduces the false-positive rate, which is one of the most common and yet difficult problems that MITE-discovery programs have tackle. The problem with clustering elements in different families when they should be together arises when doing a genome-wide search with BLAST. This can be explained because many different MITE families hit in the same location, thus giving many results that need further analysis and filtering. This scenario was very frequent when we analyzed detectMITE results. We can use the rice mPing element as an example. This element is clustered in three different families by detectMITE. When running genome wide searches using BLAST, MITE-like sequence abundance varies significantly with different values of homology and coverage.

The rule proposed in a previous study [6] of a sequence with a similarity of 80% or more in at least 80% (80-80) of the aligned sequence was too lax in our experience, especially for short elements (about 100 bp). In this case, many elements that belong to different families, according to MITE Tracker, hit exactly at the same position in the genome, despite having an identity value of 80% in VSEARCH. As a result, we used values of 95-95 homology and coverage, respectively for genome-wide searches.

### MITEs in wheat genome

To our knowledge, MITE Tracker is the first program that has been able to perform a structural search of MITEs in the whole wheat genome. In rice, 3.9% of the genome is covered with MITEs and 2.8% of the rice genes present a MITE insertion according to a previous study [35]. MITE Tracker determined that in wheat 0.16% of the wheat reference genome is covered with MITEs and that 7.01% of the genes of these reference genome contain an inserted element.

According to a search in TREP database [28], Thalos is the most abundant family with 32.99% of hits corresponding to this family, followed by the Icarus family with 17.22%. Only 5.5% of hits have no matches in TREP.

The rice genome contains a lower density of MITEs on centromeres and they are co-localized with genes [36]. Our results in wheat show that MITEs follow the same pattern as in rice. The finding of a relevant portion of genes with MITEs within its coding/regulating sequence in wheat supports the hypothesis that MITEs play an important role in gene regulation at the genome level [37]. In our study, we demonstrated that 7767 genes contain MITEs within its coding/regulating sequence (see Additional file 3: Table S2). Also, from a breeding point of view, MITEs are a valuable source of allelic variation at a gene level and marker development. Examples can be found for genes associated with wheat quality *Glu-1Bx* [38] and *Lpx-1* [39], adaptation *Vrn-1* [40] and disease resistance *Lr34* among others [41].

A point worth mentioning is also the gene regulation pathway mediated through epigenetic silencing with microRNAs and siRNAs derived from MITE elements. In rice, genes related to *gibberellin* and *brassinosteroid* homeostasis were found to be directed targeted by MITE-derived siRNAs to silence these genes and therefore affect plant height and leaf angle [42]. The development of whole genome analysis tools including the siRNA landscape, methyloma and MITEs, e.g. MITE tracker, will shed light to the molecular regulation of complex biological processes such as abiotic stress adaptation, biotic stress defense and hybrid vigor among others.

## Conclusion

Next-generation sequencing (NGS) made available high quality reference genomes of huge size such as that of the wheat genome. This made some legacy software and methods not suitable for processing this amount of data because of memory usage and processing time. MITE Tracker tackled these challenges by making use of modern clustering methods and a meticulous management of computational resources. The analysis of the rice genome unveils its performing capabilities by showing significantly better results in terms of processing time, false-positive scoring and even by discovering novel candidates to the already known elements in comparison to other software.

## Availability and requirements

Project name: MITE Tracker: an accurate method for identifying miniature inverted-repeat transposable elements in large genomes

Project home page: https://github.com/INTABiotechMJ/MITE-Tracker

Operating system(s): Platform independent

Programming language: Python

Other requirements: Python 3.6

License: GNU GPL

Any restrictions to use by non-academics: None

## Additional files

Additional file 1 Database of non-redundant MITE family database obtained from the rice genome. Wheat MITE families. Database of non-redundant MITE family database obtained from the wheat genome. (FASTA 856 kb)

Additional file 2 MITEs in several genomes. Execution summary of MITE Tracker and other tools using several genomes. (CSV 1 kb)

Additional file 3 Wheat genes. Wheat genes containing MITEs within its coding region. (CSV 1 kb)

## Abbreviations

DNA: Deoxyribonucleic acid
LCC: Local Composition Complexity
MITE: Miniature inverted-repeat transposable element
NGS: Next-generation sequencing
RNA: Ribonucleic acid
TE: Transposable element
TIR: Terminal inverted repeat
TSD: Target site duplication
